# Enhanced In Vitro Anti-Photoaging Effect of Degraded Seaweed Polysaccharides by UV/H_2_O_2_ Treatment

**DOI:** 10.3390/md21080430

**Published:** 2023-07-29

**Authors:** Wanzi Yao, Jiayu Yong, Bingxue Lv, Siyu Guo, Lijun You, Peter Chi-Keung Cheung, Viktoryia I. Kulikouskaya

**Affiliations:** 1 School of Food Science and Engineering, South China University of Technology, Guangzhou 510640, China; wanziyao22@gmail.com (W.Y.); yongjy123@163.com (J.Y.); lvbingxue@163.com (B.L.); 20231094lilith@scut.edu.cn (S.G.); 2 Research Institute for Food Nutrition and Human Health (111 Center), Guangzhou 510640, China; 3 Food & Nutritional Sciences Program, School of Life Sciences, Chinese University of Hong Kong, Hong Kong 999077, China; petercheung@cuhk.edu.hk; 4 Institute of Chemistry of New Materials, National Academy of Sciences of Belarus, 36 Skaryna Str., 220141 Minsk, Belarus; kulikouskaya@gmail.com

**Keywords:** anti-photoaging, degradation, HaCaT cells, *Sargassum fusiforme* polysaccharides, UV/H_2_O_2_

## Abstract

The high molecular weight and poor solubility of seaweed polysaccharides have limited their function and application. In this study, ultraviolet/hydrogen peroxide (UV/H_2_O_2_) treatment was used to prepare low-molecular-weight seaweed polysaccharides from *Sargassum fusiforme*. The effects of UV/H_2_O_2_ treatment on the physicochemical properties and anti-photoaging activity of *S. fusiforme* polysaccharides were studied. UV/H_2_O_2_ treatment effectively degraded polysaccharides from *S. fusiforme* (DSFPs), reducing their molecular weight from 271 kDa to 26 kDa after 2 h treatment. The treatment did not affect the functional groups in DSFPs but changed their molar percentage of monosaccharide composition and morphology. The effects of the treatment on the anti-photoaging function of *S. fusiforme* polysaccharides were investigated using human epidermal HaCaT cells *in vitro*. DFSPs significantly improved the cell viability and hydroxyproline secretion of UVB-irradiated HaCaT cells. In particular, DSFP-45 obtained from UV/H_2_O_2_ treatment for 45 min showed the best anti-photoaging effect. Moreover, DSFP-45 significantly increased the content and expression of collagen I and decreased those of pro-inflammatory cytokines, including interleukin-1β, interleukin-6, and tumor necrosis factor-α. Thus, UV/H_2_O_2_ treatment could effectively improve the anti-photoaging activity of *S. fusiforme* polysaccharides. These results provide some insights for developing novel and efficient anti-photoaging drugs or functional foods from seaweed polysaccharides.

## 1. Introduction

China has a long coastline and abundant marine macroalgal/seaweed resources with a wide range of sources. As the leading producer globally, China’s seaweed cultivation output accounts for 72% of global production, ranking first in the world [1]. But only a small portion of marine macroalgae in China is consumed fresh or undergoes simple primary processing. Most of the seaweed resources have not been exploited and utilized, resulting in a serious waste problem. Seaweed contains a variety of bioactive components, of which polysaccharides have attracted particular attention because of their diverse health-promoting functions such as anti-inflammatory [2], anti-cancer [3,4], immunomodulatory [5], and gut microbiota regulatory [6,7,8,9,10] effects. However, seaweed polysaccharides have deficiencies such as a large molecular weight, poor solubility, and low bioavailability, limiting their application [11,12]. Therefore, it is necessary to find a way to effectively degrade seaweed polysaccharides and significantly improve their solubility and biological activity, which is of great significance for exploitation and utilization.

The current methods to degrade seaweed polysaccharides mainly include physical, chemical, and biological processes. Physical methods, such as microwave, ultrasound, irradiation, and pulsed electric field, are highly efficient and environmentally friendly, but their high cost and equipment requirements limit their widespread industrial application [13,14]. Chemical methods, such as acid and alkali treatment, are easy to operate but cause environmental pollution due to their use of chemical reagents [15,16]. Biological methods, such as enzymes and microorganisms, are gentle, non-toxic, and safe, but the cost is relatively high. In addition, it is difficult to find specific enzymes and microorganisms with excellent degradation effects because of the complex structure of seaweed polysaccharides [17,18]. In recent years, the free radical degradation method, as an emerging means of polysaccharide degradation, has attracted increasing interest because of its simple operation, high efficiency, environmentally friendly nature, and low cost [19,20]. Our research group innovatively used ultraviolet/hydrogen peroxide (UV/H_2_O_2_) treatment to degrade seaweed polysaccharides and found that this method significantly reduced the molecular weight of polysaccharides and increased their solubility and bioavailability [21,22]. UV/H_2_O_2_ treatment had excellent decolorization and deproteinization effects, and the degraded polysaccharides exhibited significant anti-inflammatory activity [23].

Photoaging, also known as extrinsic aging, is a complex and multifactorial process triggered by UV irradiation. Photoaging induces oxidative stress, upregulates the expression of matrix metalloproteinases, breaks down the extracellular matrix (ECM), and causes an inflammatory response, resulting in structural, morphological, and functional changes in the skin [24]. The incidence of skin photoaging has increased over the years due to the destruction of the ozone layer and the increase in UV irradiation. How to prevent and treat UV-induced photoaging damage to the skin has become a global public health problem that needs to be solved urgently. Anti-photoaging drugs can be effective in their intended purpose, but they may also have potential side effects. For example, retinoids, which are derivatives of vitamin A, can improve skin texture and reduce wrinkles, but they can also cause skin irritation, dryness, redness, and increased sensitivity to the sun [25,26]. Compared with chemicals and synthetic drugs, natural compounds are thought to be more efficient, less toxic, with fewer side effects [27,28,29]. The strategy of using natural bioactive substances such as anthocyanin [30], peptides [31], polyphenols [32], and polysaccharides [33] to alleviate photoaging and improve skin health has been discovered as an essential method for the prevention and treatment of photoaging. Polysaccharides exert a protective and therapeutic effect on skin photoaging [34]. Our previous study showed that water-extracted polysaccharides from *S. fusiforme* exhibited a potential anti-photoaging effect [35,36]. However, these polysaccharides have a high molecular weight and poor solubility and bioavailability, which limit their further application. In addition, the anti-photoaging mechanism of *S. fusiforme* polysaccharides remains unclear.

Therefore, this study used a UV/H_2_O_2_ method to degrade *S. fusiforme* polysaccharides. The effects of UV/H_2_O_2_ treatment on the physicochemical properties and anti-photoaging activity of *S. fusiforme* polysaccharides were studied. The degraded polysaccharides with the best anti-photoaging activity were screened out, and their anti-photoaging mechanism was investigated using photoaged HaCaT cells. The results would provide some important insights for enhancing the anti-photoaging activity of polysaccharides by UV/H_2_O_2_ treatment and promote the development and utilization of marine macroalgal resources.

## 2. Results and Discussion

### 2.1. Chemical Compositions of SFP and DSFPs

The effects of UV/H_2_O_2_ treatment on the chemical composition of *S. fusiforme* polysaccharides are shown in Table 1. The results showed that the contents of total carbohydrate, reducing sugar, and sulfate of DSFPs increased gradually with the treatment time, while the content of uronic acids gradually decreased. Furthermore, the content of protein did not change significantly. The increasing contents of total carbohydrate indicated that UV/H_2_O_2_ treatment might degrade pigments and other impurities in *S. fusiforme* polysaccharides and thus improve their purity [21]. The decreasing contents of uronic acids suggested that the treatment might lead to the removal of uronic acids in polysaccharides [22]. DSFP-90 obtained by UV/H_2_O_2_ treatment for 90 min had the highest content of total carbohydrate, 72.14 ± 3.96%. DSFP-120 obtained by UV/H_2_O_2_ treatment for 120 min had the highest contents of reducing sugar and sulfate (6.16 ± 0.25% and 8.81 ± 0.50%, respectively) and the lowest contents of uronic acids (19.46 ± 1.42%).

The main reason why UV/H_2_O_2_ treatment led to changes in the chemical composition of *S. fusiforme* polysaccharides was that UV activated the decomposition of H_2_O_2_ and generated a large number of free radicals such as hydroxyl radical, peroxy radical, and hydrogen radical, which then attacked the structure of polysaccharides, causing changes in their chemical composition [37,38,39]. The increase in the content of reducing sugar may be attributed to the increase in reducing sugar terminals caused by the breakage of glycosidic bonds during the treatment. The decrease in the content of uronic acids might be caused by the removal of uronic acids during oxidative degradation. The increase in the content of sulfate might be related to the absence of free radical attack on the sulfate group linking sites in polysaccharides [22].

### 2.2. Molecular Weights and Yields of SFP and DSFPs

**Figure 1:**
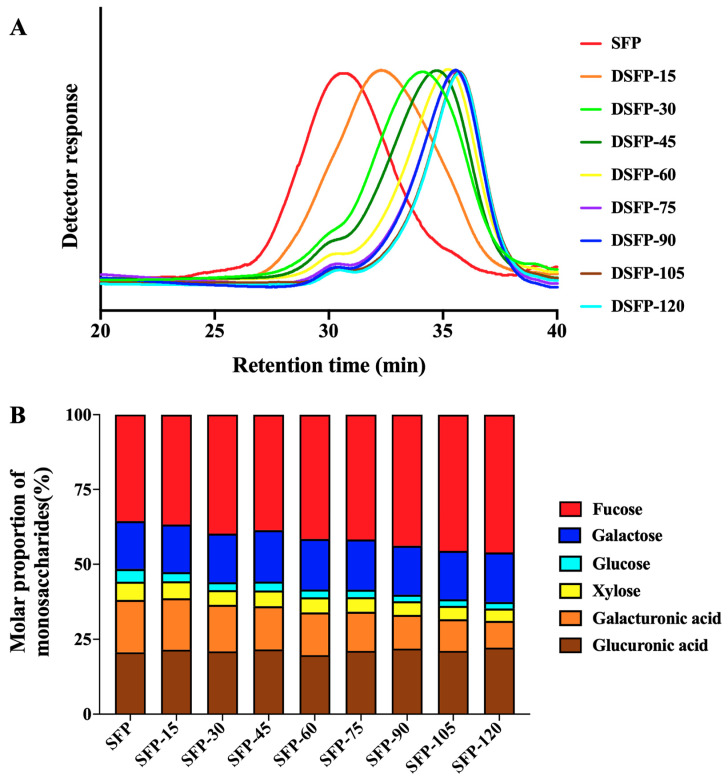
Molecular weights (**A**) and monosaccharide compositions (**B**) of SFP and DSFPs. Note: DSFPs include DSFP-15, DSFP-30, DSFP-45, DSFP-60, DSFP-75, DSFP-90, DSFP-105, and DSFP-120.

The effects of UV/H_2_O_2_ treatment on the molecular weight of *S. fusiforme* polysaccharides are shown in Table 2 and Figure 1A. After the treatment, the molecular weights of DSFPs were significantly reduced compared with that of SFP (271 kDa), and the decrease in molecular weight gradually increased with the treatment time. The molecular weights of DSFP-15, DSFP-30, DSFP-45, DSFP-60, DSFP-75, DSFP-90, DSFP-105, and DSFP-120 were 128, 59, 43, 36, 31, 28, 28, and 26 kDa, respectively. UV/H_2_O_2_ treatment effectively degraded *S. fusiforme* polysaccharides, most likely because the free radicals generated by UV-excited H_2_O_2_ decomposition attacked the structure of polysaccharides, causing their degradation and reducing their molecular weight. And as time went on, more free radicals were created, which increased the degree of glycosidic bond cleavage.

The yields of DSFPs shown in Table 2 reduced gradually with the treatment time. The yield of DSFP-15 obtained by UV/H_2_O_2_ treatment for 15 min was 65.7%, while the yield of DSFP-120 obtained by UV/H_2_O_2_ treatment for 120 min was 30.0%. This might be because the degraded polysaccharides were purified by dialysis after treatment. As the degradation time increased, *S. fusiforme* polysaccharides were broken down more finely, and more low molecular weight fragments were removed during dialysis, resulting in a gradual decrease in yield.

### 2.3. Monosaccharide Compositions of SFP and DSFPs

The effects of UV/H_2_O_2_ treatment on the monosaccharide composition of *S. fusiforme* polysaccharides are shown in Table 3 and Figure 1B. The results showed that seven monosaccharides were detected in SFP. Fucose, the most abundant monosaccharide of SFP, accounted for 35.58% of the total, followed by glucuronic acid, galacturonic acid, galactose, glucose, and xylose with respective amounts of 20.62%, 17.53%, 15.96%, 6.12%, and 4.19%. DSFPs had the same type of monosaccharide composition as SFP, indicating that UV/H_2_O_2_ treatment did not change the type of monosaccharide composition of *S. fusiforme* polysaccharides, which is consistent with the previous studies [40]. However, there were considerable changes in the molar percentages of their monosaccharide composition. With the increase in treatment time, the molar percentages of fucose increased steadily from 35.58% to 46.04%. The molar percentages of glucose, xylose, and galacturonic acid decreased gradually, from 4.19% to 2.20%, 6.12% to 4.11%, and 17.53% to 8.95%, respectively. The findings suggested that glucose, xylose, and galacturonic acid residues might be the main attach sites of free radicals during the treatment. The lack of a clear pattern in the molar percentages of the other monosaccharides might be attributed to the complex structure of *S. fusiforme* polysaccharides and the non-selectivity of free radicals’ attack sites.

### 2.4. Scanning Electron Microscope (SEM) Analysis

**Figure 2:**
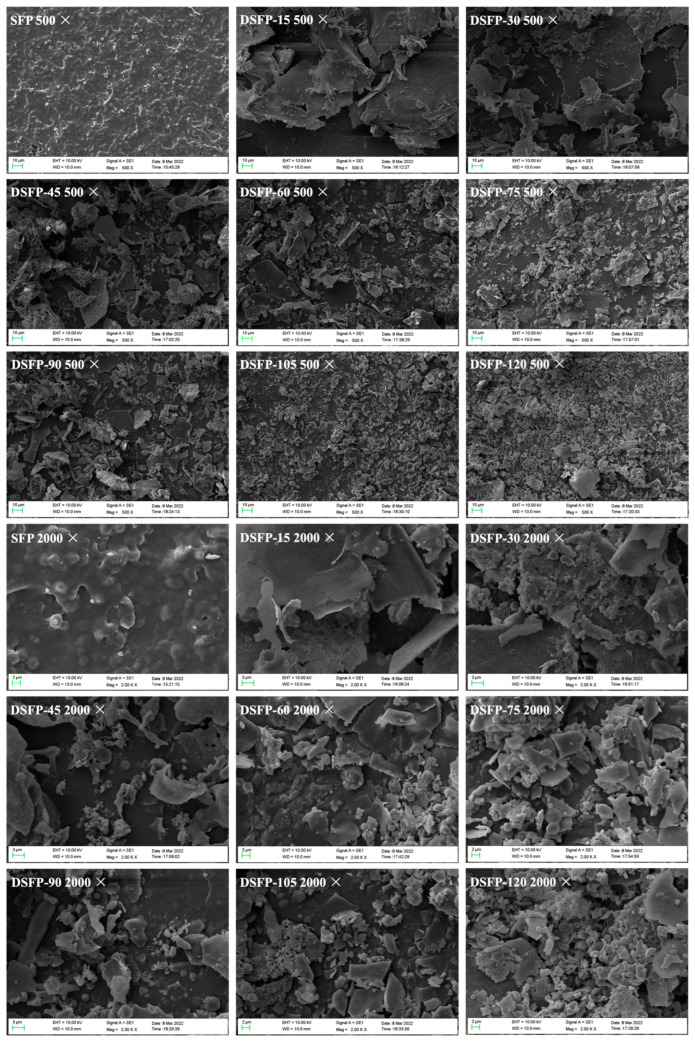
SEM spectra of SFP and DSFPs. Note: DSFPs include DSFP-15, DSFP-30, DSFP-45, DSFP-60, DSFP-75, DSFP-90, DSFP-105, and DSFP-120.

The effects of UV/H_2_O_2_ treatment on the surface morphology and microstructure of *S. fusiforme* polysaccharides are shown in Figure 2. SFP had a tight structure with a rough and uneven surface, exhibiting a thick sheet-like morphology. The structure of SFP was dense and compact, revealing that their sugar chains were tightly aggregated. After UV/H_2_O_2_ treatment, the apparent morphology of polysaccharides changed obviously. Although DSFP-15 obtained by the treatment for 15 min still displayed a sheet structure, its surface roughness and compactness were significantly reduced. In addition, the slight fragmentation of polysaccharides was observed, possibly due to the breakage of glycosidic bonds during UV/H_2_O_2_ treatment. DSFP-30 obtained by the treatment for 30 min still showed a flake-like structure with a smooth surface, but the fragmentation of DSFP-30 was more than that of DSFP-15. When the treatment time reached 45 min, the product DSFP-45 showed a broken and finely fragmented structure, and its surface changed from dense and tightly aggregated to a loosened form. When the treatment duration approached 60 min, the surface morphology of DSFP-60 was disrupted, and severe fragmentation of polysaccharides was observed. When the treatment time reached 105 min, the surface of DSFP-105 was destroyed, and many small irregular fragments were formed and aggregated together.

UV/H_2_O_2_ treatment disrupted the structure of *S. fusiforme* polysaccharides and caused significant changes in their apparent morphology. The surface of polysaccharides became finely crumbled, and the degree of fragmentation was positively correlated with the treatment time. These changes may be because the free radicals generated by UV/H_2_O_2_ treatment attacked the structure of polysaccharides, leading to the breakage of covalent bonds between polysaccharides, such as the glycosidic bonds connecting monosaccharides, which in turn reduced the size of particles, the degree of aggregation, and the intermolecular force of polysaccharides [41,42]. The main chain and substituent groups of polysaccharides were more easily degraded, and the degree of molecular crosslinking was reduced, eventually leading to significant changes in the morphology of polysaccharides [43].

### 2.5. Fourier Transform Infrared Spectroscopy (FT-IR) Spectra Analysis

**Figure 3:**
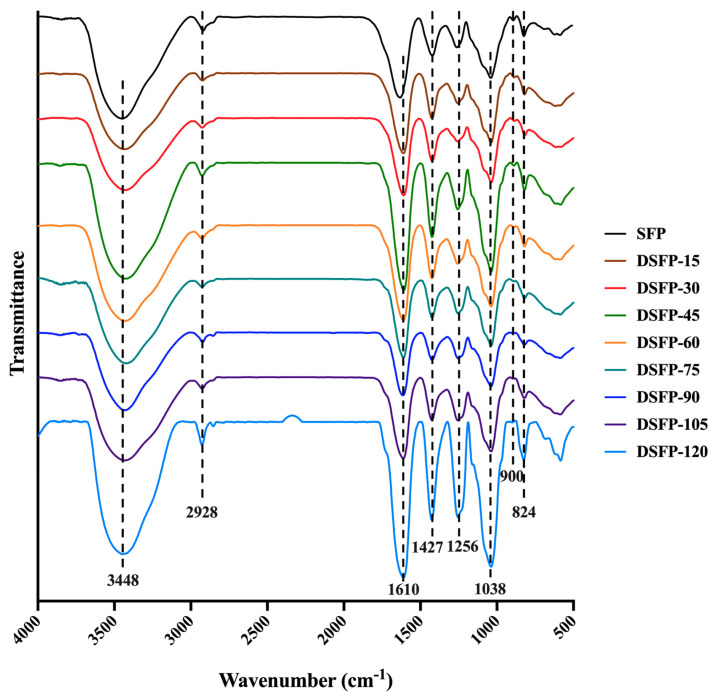
FT-IR spectra of SFP and DSFPs. Note: DSFPs include DSFP-15, DSFP-30, DSFP-45, DSFP-60, DSFP-75, DSFP-90, DSFP-105, and DSFP-120.

The effects of UV/H_2_O_2_ treatment on the main functional groups of *S. fusiforme* polysaccharides are shown in Figure 3. The FT-IR spectra showed that SFP and DSFPs had similar characteristic absorption peaks, indicating that the UV/H_2_O_2_ treatment did not change the major functional group types of polysaccharides. The broad absorption peak at 3448 cm^−1^ was a typical O–H stretching vibration, and the weak peak at 2928 cm^−1^ was attributed to a C–H stretching vibration, both of which are characteristic absorption peaks of polysaccharides [44]. The strong absorption peak at 1610 cm^−1^ was attributed to a C=O stretching vibration, and the absorption peak at 1427 cm^−1^ was due to C–O bending, indicating the existence of uronic acids [45], which was consistent with the results of chemical composition determination. The absorption band at 1256 cm^−1^ was ascribed to a S=O stretching vibration, and the weak absorption peak at 824 cm^−1^ was due to a C–O–S stretching vibration, revealing the presence of sulfate [46]. The strong signal at 1038 cm^−1^ was generated by a C–O–H stretching vibration, which was the characteristic peak of the pyranose ring [47]. The weak absorption peak at 900 cm^−1^ was the characteristic of β-type glycosidic linkages [44]. These results showed that SFP and DSFPs were typical sulfated polysaccharides containing pyranose rings and β-type glycosidic linkages. Therefore, UV/H_2_O_2_ treatment might only cleave the glycosidic bonds of polysaccharides without affecting most of the functional groups.

### 2.6. Effects of SFP and DSFPs on the Cell Viability of Photoaged HaCaT Cells

Photoaging is the external aging of human skin caused by UV exposure. When human skin is exposed to UVB light, UVB irradiation directly damages skin cells and destroys the structure and function of skin cells, resulting in skin photoaging [48]. In this study, HaCaT cells were pretreated with different concentrations of SFP and DSFPs. The cell viability of UVB-irradiated HaCaT cells was determined by the MTT method to investigate the in vitro anti-photoaging effects of SFP and DSFPs.

**Figure 4:**
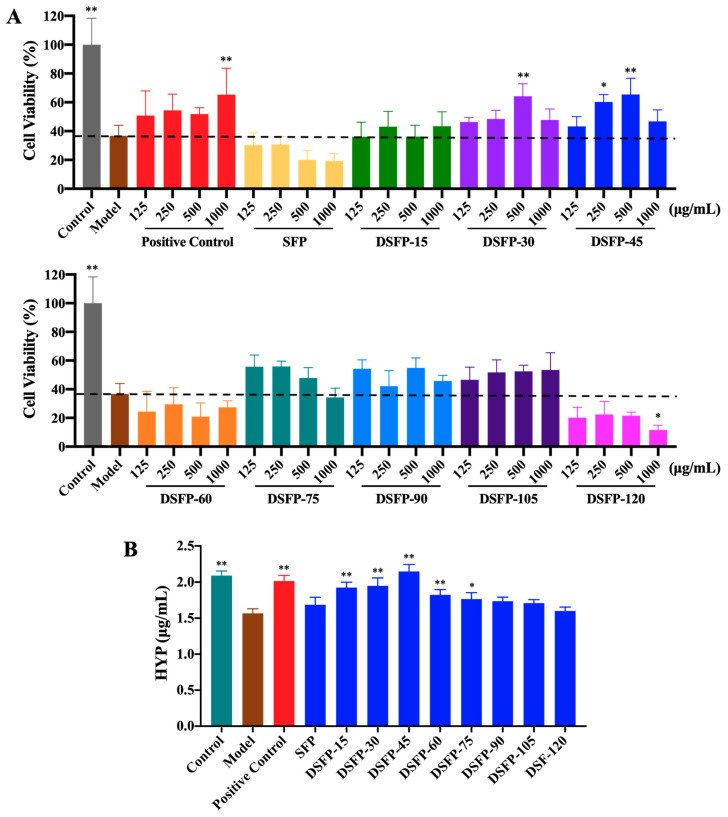
Effects of SFP and DSFPs on cell viability (**A**) and the level of HYP (**B**) of photoaged HaCaT cells. Note: DSFPs include DSFP-15, DSFP-30, DSFP-45, DSFP-60, DSFP-75, DSFP-90, DSFP-105, and DSFP-120. * *p* < 0.05 or ** *p* < 0.01 vs. Model group.

As shown in Figure 4A, after UVB irradiation, the cell viability of HaCaT cells in the Model group was significantly lower (*p* < 0.01) than that in the Control group, indicating that the cells were damaged by photoaging. The cell viability of HaCaT cells treated with hyaluronic acid, DSFP-30, and DSFP-45 significantly increased, revealing that the above three treatments all had significant anti-photoaging activity. Among them, DSFP-45 obtained from UV/H_2_O_2_ treatment for 45 min showed the best anti-photoaging effect on UVB-irradiated HaCaT cells, exhibiting significant anti-photoaging activity when its concentration was 250 μg/mL (*p* < 0.05). When the treated concentration of DSFP-30 and DSFP-45 reached 1000 μg/mL, the cell viability of HaCaT cells decreased, which may be due to the cytotoxicity induced by increased byproducts from oxidative degradation. SFP without UV/H_2_O_2_ treatment did not significantly increase the cell viability of photoaged HaCaT cells, indicating that UV/H_2_O_2_ treatment improved the in vitro anti-photoaging activity of *S. fusiforme* polysaccharides. DSFP-30 and DSFP-45 showed the strongest anti-photoaging effect at a concentration of 500 μg/mL. Therefore, the concentration of 500 μg/mL was selected as the test concentration for the following study.

### 2.7. Effects of SFP and DSFPs on the Level of Hydroxyproline (HYP)

HYP, a unique amino acid in collagen, is one of the main components of collagen tissue, accounting for 14% of collagen. HYP is an indicator of connective tissue synthesis and reflects collagen metabolism in connective-tissue diseases [49]. The level of HYP secreted by skin cells reflects the collagen synthesis and metabolism of skin cells. It can be used to screen anti-photoaging substances. In this study, HaCaT cells were pretreated with SFP and DSFPs at a concentration of 500 μg/mL. The HYP content of UVB-irradiated cells was measured to investigate the in vitro anti-photoaging activity of SFP and DSFPs.

As shown in Figure 4B, the HYP level of HaCaT cells in the Model group was significantly lower (*p* < 0.01) than that in the Control group, suggesting that the collagen synthesis of cells was inhibited by UVB irradiation. The HYP contents of HaCaT cells treated with hyaluronic acid, DSFP-15, DSFP-30, DSFP-45, DSFP-60, and DSFP-75 significantly increased, indicating that they all promoted collagen synthesis and metabolism of skin cells and exhibited significant anti-photoaging effects. Among them, DSFP-45 showed the best anti-photoaging activity, with an increase of 36.94% in the amount of HYP (2.15 μg/mL) compared to the Model group (1.57 μg/mL). SFP without UV/H_2_O_2_ treatment did not significantly increase the HYP level of photoaged HaCaT cells, indicating that the treatment could improve the anti-photoaging activity of *S. fusiforme* polysaccharides. UV/H_2_O_2_ treatment effectively degraded the polysaccharides, resulting in the formation of low molecular weight polysaccharides. This reduction in molecular weight potentially improved their bioavailability, solubility, and cellular uptake, enhancing their interaction with target cells and increasing their bioactivity [50,51]. Besides, UV/H_2_O_2_ treatment did not significantly alter the functional groups in DSFPs, ensuring that the essential chemical structures responsible for their biological effects remained intact. This preservation of functional groups facilitated their interaction with cellular receptors, signaling pathways, and target molecules involved in photoaging processes [52]. The results of both Section 3.6 and Section 3.7 showed that DSFP-45 obtained from UV/H_2_O_2_ treatment for 45 min had the strongest in vitro anti-photoaging activity. Therefore, DSFP-45 was selected for the subsequent study.

### 2.8. Effects of SFP and DSFP-45 on the Level and Expression of Collagen I

Collagen I, also known as type I collagen, is the most abundant collagen protein in the human body and also the most abundant structural protein in connective tissues such as skin, bones, ligaments, and tendons [53]. It belongs to the family of fibrillar collagens and is a major component of the ECM. It is a procollagen molecule that mainly consists of two structures: a heterotrimer composed of two α1 chains and one α2 chain and a homotrimer composed of three identical α1 chains [54]. The content of collagen I secreted by skin cells reflects the synthetic metabolism of the ECM and can be used as an important evaluation index of photoaging. In this study, HaCaT cells were pretreated with SFP and DSFP-45 (125, 250, and 500 μg/mL). The content of pro-collagen I α1 and the expression level of collagen I of UVB-irradiated cells were determined to investigate the in vitro anti-photoaging mechanisms of *S. fusiforme* polysaccharides.

**Figure 5:**
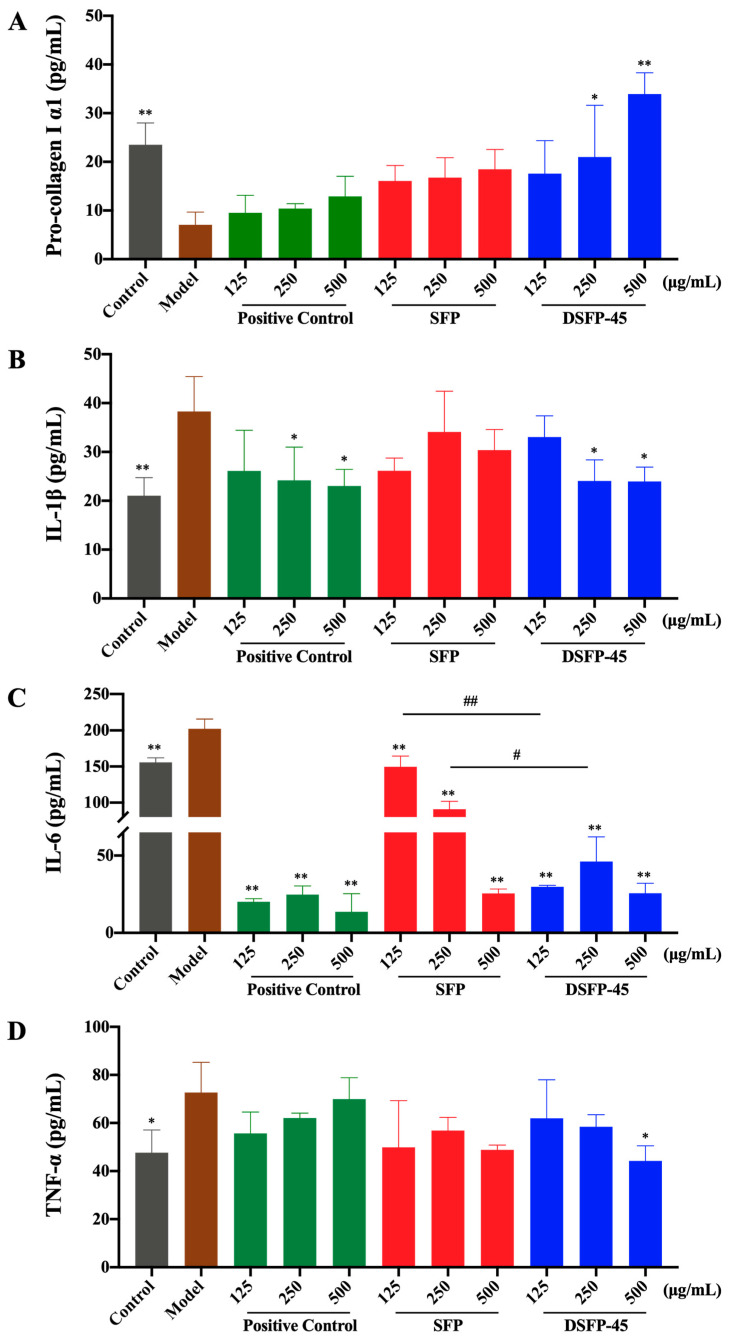
Effects of SFP and DSFP-45 on the levels of pro-collagen I α1 (**A**), IL-1β (**B**), IL-6 (**C**), and TNF-α (**D**) of photoaged HaCaT cells. * *p* < 0.05 or ** *p* < 0.01 vs. Model group. # *p* < 0.05 or ## *p* < 0.01 vs. SFP group.

**Figure 6:**
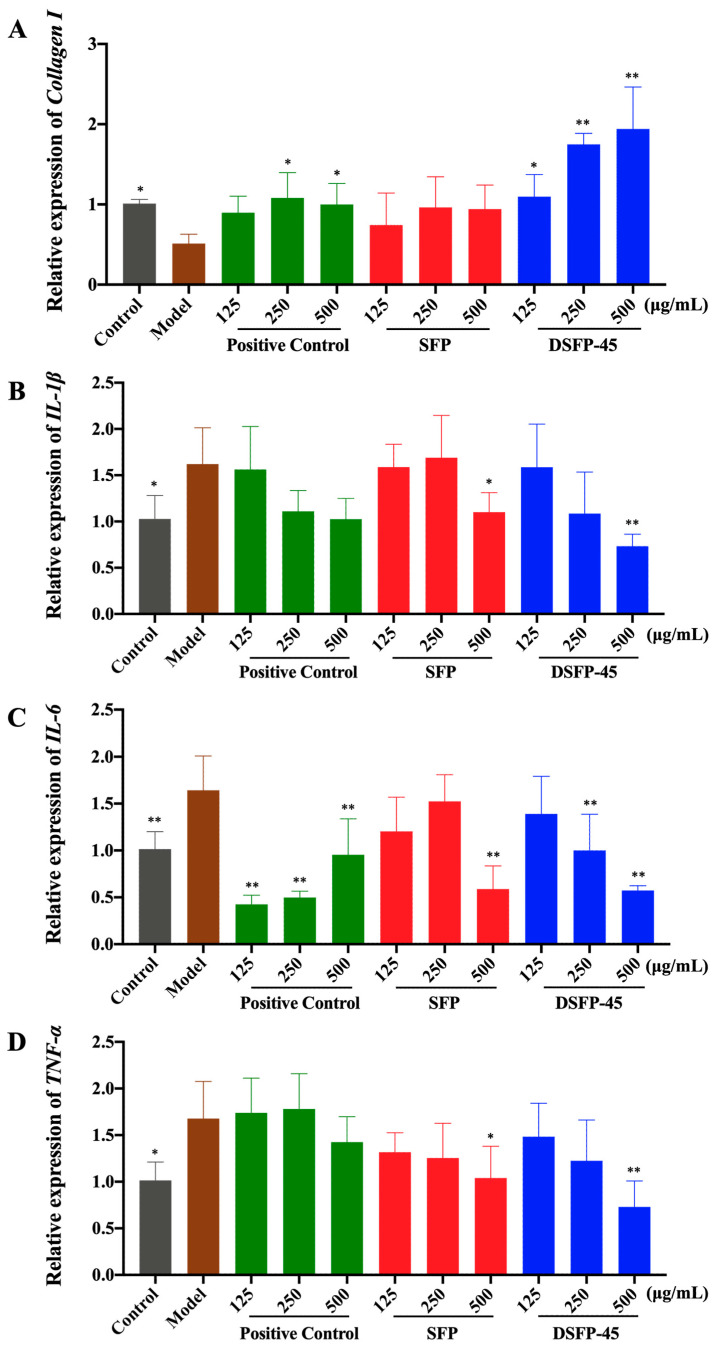
Effects of SFP and DSFP-45 on the expression of *collagen I* (**A**), *IL-1β* (**B**), *IL-6* (**C**), and *TNF-α* (**D**) of photoaged HaCaT cells. * *p* < 0.05 or ** *p* < 0.01 vs. Model group.

As shown in Figure 5A, after UVB radiation, the content of pro-collagen I α1 in the Model group significantly decreased compared with that in the Control group (*p* < 0.01), indicating that UVB radiation induced collagen degradation. The level of pro-collagen I α1 of HaCaT cells treated with hyaluronic acid and SFP slightly increased but did not have significant difference from that in the Model group. The pro-collagen I α1 level of the cells treated with DSFP-45 significantly increased (*p* < 0.05). When the intervention concentration of DSFP-45 reached 500 μg/mL, the pro-collagen I α1 (33.93 pg/mL) increased 3.80 times compared to the Model group (7.07 pg/mL). As shown in Figure 6A, the expression level of *collagen I* in the Model group was significantly lower than that in the Control group (*p* < 0.05). DSFP-45 significantly upregulated the expression of *collagen I* in HaCaT cells (*p* < 0.05). Under the intervention of DSFP-45 at 125, 250, and 500 μg/mL, the relative expression levels of *collagen I* in HaCaT cells increased by 1.14 times, 2.42 times, and 2.80 times compared with those in the Model group. DSFP-45 increased the content and expression of collagen I, and this behavior was concentration-dependent, indicating that DSFP-45 exerted an anti-photoaging effect by promoting the synthesis of ECM. SFP without UV/H_2_O_2_ treatment did not significantly increase the content and expression of collagen I. Therefore, UV/H_2_O_2_ treatment for 45 min improved the antagonistic effect of *S. fusiforme* polysaccharides on skin damage.

### 2.9. Effects of SFP and DSFP-45 on the Levels and Expression of Pro-Inflammatory Cytokines

UV radiation can trigger a cellular inflammatory response by stimulating the release of cytokines, chemokines, and other inflammatory mediators in the epidermis [55]. The release of pro-inflammatory cytokines increases the permeability of cell capillaries, leads to the infiltration and activation of inflammatory cells such as macrophages and neutrophils, causes inflammatory damage to the skin, and ultimately results in skin photoaging [56]. Studies have shown that UV irradiation can induce the release of pro-inflammatory cytokines such as interleukin-1β (IL-1β), interleukin-6 (IL-6), and tumor necrosis factor-α (TNF-α) by keratinocytes, thereby disrupting collagen synthesis and interfering with ECM metabolism [57]. Pro-inflammatory cytokines can induce extracellular regulatory protein kinase pathway phosphorylation, promote matrix collagen degradation, and ultimately lead to cellular photoaging damage [58]. Moreover, pro-inflammatory cytokines also affect the function of Langerhans cells as antigen-presenting cells by reducing the number of Langerhans cells in the skin, increasing the tolerance of T lymphocytes, inhibiting the immune function of the body, weakening the body’s resistance to photoaging and aggravating photodamage [59]. In this study, HaCaT cells were pretreated with SFP and DSFP-45 (125, 250, and 500 μg/mL). The amounts and the expression levels of IL-1β, IL-6, and TNF-α of photoaged cells were measured to explore the mechanisms of the anti-photoaging action of *S. fusiforme* polysaccharides.

As shown in Figure 5B–D, the levels of IL-1β, IL-6, and TNF-α in the Model group were significantly higher than those in the Control group (*p* < 0.05), indicating that UVB radiation induced an inflammatory response in HaCaT cells. The amounts of IL-1β of cells treated with hyaluronic acid and DSFP-45 significantly decreased compared with the Model group (*p* < 0.05), demonstrating that hyaluronic acid and DSFP-45 inhibited the ability of cells to secrete IL-1β. The content of IL-1β of cells treated with SFP decreased slightly, but there was no significant difference, suggesting that UV/H_2_O_2_ treatment for 45 min could enhance the anti-inflammatory activity of *S. fusiforme* polysaccharides. After treatment with hyaluronic acid, SFP, and DSFP-45, the levels of IL-6 of HaCaT cells all significantly decreased (*p* < 0.01), indicating that hyaluronic acid, SFP, and DSFP-45 could inhibit the secretion of IL-6 and had significant anti-inflammatory activity. But the inhibitory effect of DSFP-45 on IL-6 secretion was better than that of SFP. Moreover, the content of TNF-α of the cells treated with DSFP-45 at 500 μg/mL significantly decreased when compared to the Model group (*p* < 0.05). The levels of TNF-α secreted by HaCaT cells treated with hyaluronic acid and SFP decreased slightly, but there was no significant difference. These results indicated that DSFP-45 exerted anti-photoaging effects by inhibiting the secretion of pro-inflammatory cytokines and reducing the damage of cellular inflammation. In addition, the effect of DSFP-45 was superior to that of SFP, indicating that UV/H_2_O_2_ treatment for 45 min improved the inhibitory effect of *S. fusiforme* polysaccharides on the cellular inflammatory response.

As shown in Figure 6B–D, the expression levels of *IL-1β*, *IL-6*, and *TNF-α* in the Model group were significantly higher than those in the Control group (*p* < 0.05), indicating that UVB irradiation triggered a cellular inflammatory response. Compared with the Model group, DSFP-45 significantly downregulated the expression levels of *IL-1β*, *IL-6*, and *TNF-α* in HaCaT cells (*p* < 0.05), with the regulatory effects showing concentration dependence. Under the intervention of DSFP-45 at doses of 125, 250, and 500 μg/mL, the relative expression levels of *IL-1β* in HaCaT cells decreased by 2.01%, 32.96%, and 54.73% compared with those in the Model group, respectively. The relative expressions of *IL-6* declined by 15.33%, 39.09%, and 65.13%, respectively. The relative expressions of *TNF-α* dropped by 11.56%, 26.94%, and 56.50%, respectively. SFP without UV/H_2_O_2_ treatment also exhibited anti-photoaging activity at high doses. SFP significantly downregulated the expression of *IL-1β*, *IL-6*, and *TNF-α* (*p* < 0.05) at a concentration of 500 μg/mL, indicating that SFP also inhibited the cellular inflammatory response, demonstrating anti-photoaging activity. But SFP was not as effective in exerting anti-inflammatory effects as DSFP-45. Thus, UV/H_2_O_2_ treatment for 45 min significantly enhanced the in vitro anti-photoaging activity of *S. fusiforme* polysaccharide.

Many reports have shown the anti-inflammatory effects of seaweed polysaccharides in a UVB radiation-induced photoaging model. For example, a fucoidan isolated from the brown seaweed *Turbinaria ornata* attenuated UVB irradiation-induced in vivo photodamage via inhibiting oxidative stress and inflammatory response in zebrafish [60]. *Gracilaria lemaneiformis* polysaccharides inhibited the UVB-induced inflammatory response by restraining the upregulation of iNOS (UVB-induced inflammation marker) and suppressing the expression of P-ERK and NF-κB [61]. A low-molecular-weight fucoidan from *Ecklonia cava* exerted anti-photoaging effects by decreasing IL-1β and increasing IL-10 levels [62]. These studies provide a theoretical basis for using natural seaweed polysaccharides as anti-photoaging drugs or functional foods. Therefore, DSFP-45 has the potential to serve as an attractive candidate for therapeutic interventions against skin aging, particularly photoaging caused by UV radiation.

## 3. Materials and Methods

### 3.1. Materials and Chemicals

*S. fusiforme* was harvested (August 2020) from Wenzhou, Zhejiang Province, China. The collected seaweed samples were washed, sundried, and crushed into a fine powder using a grinder (FW135, Tianjin Taisite Instrument Co., Ltd., Tianjin, China) and shifted with a 40-mesh standard sieve. The fine powder was ground into ultra-micro powder using an ultra-micro pulverizer (XDW-6BI, Jinan Tatsu Micro Machinery Co. Ltd., Jinan, Shandong Province, China).

Dextran standards (4.66, 12.6, 63.3, 126, and 556 kDa) and monosaccharide standards (glucose, fucose, galactose, arabinose, xylose, fructose, galacturonic acid, and glucuronic acid) were purchased from Sigma-Aldrich Chemical Co. (St. Louis, MO, USA). Dulbecco’s Modified Eagle’s Medium (DMEM), penicillin-streptomycin, fetal bovine serum (FBS), phosphate-buffered saline (PBS), trypsin-ethylenediaminetetraacetic acid, and other reagents for cell culture were purchased from Gibco Biotechnology Co., Ltd. (Grand Island, NY, USA). Primers of genes were obtained from Sangon Biotech Co., Ltd. (Shanghai, China). All other reagents and chemicals used were of analytical grade. 

### 3.2. Preparation and Degradation of S. fusiforme Polysaccharides

*S. fusiforme* crude polysaccharides (named SFP) were prepared using our previously reported method [63]. SFP was further degraded by UV/H_2_O_2_ treatment. SFP was dissolved in distilled water, and H_2_O_2_ was added, resulting in a final SFP concentration of 2.5 mg/mL and a final H_2_O_2_ concentration of 100 mM. Then, the solution was treated by UV radiation (HOPE-MED 8140, Tianjin Hepu Industry & Trade Co. Ltd., Tianjin, China) at an average irradiation power of 950 μW/cm^2^ for 15, 30, 45, 60, 75, 90, 105, and 120 min, respectively. After degradation, manganese dioxide (MnO_2_) was added immediately and stirred on a magnetic stirrer for 12 h to remove the residual H_2_O_2_. Then, the solution was concentrated at 60 °C to 1/5 of its initial volume by a reduced rotary evaporator (Hei-VAP Value Digital, Heidoph, Nuremberg, Germany). MnO_2_ was removed by centrifugation (12,000× *g*, 5 min), and the supernatant was purified by dialysis (3 kDa molecular weight cut-off) for 48 h. Finally, the retentate was collected, concentrated by evaporation, and lyophilized by vacuum freeze-drying (Alpha 1–2 LD Plus, Martin Christ Gefriertrocknungsanlagen GmbH, Osterode am Harz, Germany) to obtain degraded products, named DSFP-15, DSFP-30, DSFP-45, DSFP-60, DSFP-75, DSFP-90, DSFP-105, and DSFP-120, respectively. The yield of degraded polysaccharides was calculated according to the following equation:

$$\mathrm{Yield}\;\left(\%\right)=\frac{m_{x}}{m_{0}}\times 100$$

where *m*_0_ was the mass of dry SFP (g) and *m_x_* was the mass of dry DSFPs (g).

### 3.3. Determination of Chemical Composition of S. fusiforme Polysaccharides

The content of total carbohydrate was determined by the phenol-sulfuric acid method using fucose as the standard [64] since fucose was the most predominant monosaccharide in *S. fusiforme* polysaccharides [21,35]. The content of protein was measured using the Total Protein Assay Kit (A045-4, Nanjing Jiancheng Bioengineering Institute, Nanjing, China) according to the manufacturer’s protocol. The amount of reducing sugar was determined by the dinitrosalicylic acid method with glucose as the standard [65]. The content of uronic acids was measured using the carbazole-sulfuric acid method with glucuronic acid as the reference [66], since glucuronic acid was the most abundant uronic acid in *S. fusiforme* polysaccharides [67]. The content of sulfate was measured by the barium sulfate turbidimetry method using potassium sulfate as the standard [68].

### 3.4. Determination of Molecular Weight of S. fusiforme Polysaccharides

The molecular weight of polysaccharides was measured by high-performance gel permeation chromatography (HPGPC). The HPGPC system (Shimadzu LC-20A, Shimadzu Instrument Co., Ltd., Tokyo, Japan) was equipped with a TSK-GEL G-3000PWXL column (7.8 mm × 300 mm i.d., 7 μm, Tosoh Corporation, Tokyo, Japan) and a TSK-GEL G-6000 PWXL column (7.8 mm × 300 mm i.d.,13 μm, Tosoh Corporation, Tokyo, Japan) that were linked in series, eluted with 0.02 M potassium dihydrogen phosphate (KH_2_PO_4_) at a flow rate of 0.5 mL/min and detected by a Waters 2414 differential refractive index detector (Waters Co. Ltd., Milford, MA, USA). The column temperature was kept at 35 ± 1 °C. The polysaccharides were dissolved in 0.02 M KH_2_PO_4_ and filtered through a 0.22 µm filter membrane. An aliquot of 25 μL of the sample was injected into the system. The molecular weight of polysaccharides was determined by the calibration curve made from dextran standards with known molecular weights (4.66, 12.6, 63.3, 126, and 556 kDa).

### 3.5. Determination of Monosaccharide Composition of S. fusiforme Polysaccharides

The monosaccharide composition of polysaccharides was determined by ion chromatography (IC). The IC system (Dionex ICS-3000, Dionex Corp. Sunnyvale, CA, USA) was equipped with a CarboPac^TM^ PA20 column (3 mm × 150 mm), eluted with water/NaOH at a flow rate of 0.5 mL/min, and detected with an electrochemical detector. The column temperature was maintained at 30 °C. The polysaccharides were hydrolyzed with 2 M trifluoroacetic acid at 105 °C for 6 h. The remaining acid was removed by evaporation under reduced pressure at 60 °C, and the methanol was added to dissolve the hydrolysate fully and evaporated again. The above step was repeated 5 times to obliterate the residual trifluoroacetic acid. The hydrolyzed product was re-dissolved in ultrapure water and filtered through a 0.22 µm filter membrane. An aliquot of 15 μL of the sample was injected into the system. The content of each monosaccharide in polysaccharides was calculated by reference to the calibration curve made from monosaccharide standards (fucose, rhamnose, arabinose, galactose, glucose, xylose, mannose, galacturonic acid, and glucuronic acid).

### 3.6. SEM Analysis of S. fusiforme Polysaccharides

The morphology and microstructure of polysaccharides were characterized by SEM (EVO 18, Carl Zeiss AG, Oberkohen, Baden-Wurttemberg, Germany). The lyophilized polysaccharides were coated with a thin film of gold, placed on the electron microscope stage, and observed under vacuum at an acceleration voltage of 10 kV. Micrographs were taken at magnifications of 500× and 2000×.

### 3.7. FT-IR Spectra Analysis of S. fusiforme Polysaccharides

The functional group type of polysaccharides was measured by FT-IR. Dried polysaccharides were crushed with potassium bromide powder, pressed into a pellet, and detected in a wavelength range from 4000 cm^−1^ to 500 cm^−1^ onto a FT-IR spectrophotometer (Tensor 27, Bruker Co. Ltd., Bergisch Gladbach, Germany).

### 3.8. Anti-Photoaging Activity of S. fusiforme Polysaccharides

#### 3.8.1. Cell Culture and UVB Irradiation

Human immortalized keratinocyte cell line (HaCaT) was obtained from Cell Resource Center, IBMS, CAMS/PUMC (Beijing, China). HaCaT cells were cultured in DMEM containing 10% (*v*/*v*) FBS, 100 units/mL penicillin, and 100 μg/mL streptomycin at 37 °C in a humidified incubator with 5% CO_2_. UVB irradiation was performed according to the method previously reported by our group with some modifications [36].

#### 3.8.2. Determination of Cell Viability

The effects of SFP and DSFPs (DSFP-15, DSFP-30, DSFP-45, DSFP-60, DSFP-75, DSFP-90, DSFP-105, and DSFP-120) on cell viability of photoaged HaCaT cells were measured using 3-(4,5-dimethylthiazol-2-yl)-2,5-diphenyltetrazolium bromide (MTT) assay. HaCaT cells were cultured for 24 h, washed with PBS, trypsinized with trypsin-EDTA, and seeded in 96-well plates at 2 × 10^4^ cells per well. After incubating at 37 °C for 12 h, the medium was discarded, and the cells were washed with PBS and incubated for another 12 h with serum-free DMEM. Then, the cells were cultured in the serum-free medium with various concentrations (125, 250, 500, and 1000 μg/mL) of hyaluronic acid (Positive Control), SFP, and DSFPs at 37 °C for 24 h. After removing the medium, the cells were covered with PBS and exposed to 3 mJ/cm^2^ of UVB irradiation in a skin photoaging apparatus (HOPE-MED 8140, Tianjin Hepu Industry & Trade Co. Ltd., Tianjin, China). After UVB irradiation, PBS was replaced with DMEM, and the cells were incubated at 37 °C for 24 h. After removing the culture medium, the cell viability was measured using the MTT Cell Proliferation and Cytotoxicity Assay Kit (G020-1, Nanjing Jiancheng Bioengineering Institute, Nanjing, China) according to the manufacturer’s protocol. Wells without cells acted as the Blank group. Wells with cells but without UVB and drug exposure served as the Control group. Wells with cells and UVB exposure but without drug treatment acted as the Model group. The absorbance was measured at 570 nm using a microplate reader (SpectraMax 190, Molecular Devices, Sunnyvale, CA, USA). The absorbance of the Blank group was subtracted from all samples. The absorbance of the Control group was defined as 100% live cells.

#### 3.8.3. Determination of HYP Level

The effects of SFP and DSFPs (DSFP-15, DSFP-30, DSFP-45, DSFP-60, DSFP-75, DSFP-90, DSFP-105, and DSFP-120) on the HYP level of photoaged HaCaT cells were measured using HYP assay. HaCaT cells were seeded in 12-well plates at a concentration of 2.5 × 10^5^ cells per well and treated according to Section 3.8.2. The cell-free culture supernatant was collected to measure the level of HYP using the HYP Assay Kit (A030-1-1, Nanjing Jiancheng Bioengineering Institute, Nanjing, China) in accordance with the manufacturer’s protocol.

#### 3.8.4. Measurement of Pro-Collagen I α1 Content of HaCaT Cells

The effects of SFP and DSFP-45 on the pro-collagen I α1 content of photoaged HaCaT cells were studied by enzyme linked immunosorbent assay (ELISA). HaCaT cells were incubated in 6-well plates at a concentration of 6 × 10^5^ cells per well and treated following Section 3.8.2. The cell-free culture supernatant was collected to measure the amount of pro-collagen I α1 using the Human Pro-collagen I α1 ELISA Kit (EHC083aQT.96, NeoBioscience Technology Co., Ltd., Shenzhen, China) according to the manufacturer’s instructions.

#### 3.8.5. Measurement of Pro-Inflammatory Cytokines

The effects of SFP and DSFP-45 on the levels of pro-inflammatory cytokines, including IL-1β, IL-6, and TNF-α, were studied by ELISA. The level of IL-1β was measured using the Human IL-1β ELISA Kit (EHC002b.48, NeoBioscience Technology Co., Ltd., Shenzhen, China) according to the manufacturer’s protocol. The content of IL-6 was determined using the Human IL-6 ELISA Kit (70-EK106/2-96, Multi Sciences Biotech Co., Ltd., Hangzhou, China). The amount of TNF-α was detected by the Human TNF-α ELISA Kit (70-EK182-96, Multi Sciences Biotech Co., Ltd., Hangzhou, China).

#### 3.8.6. Quantitative Reverse Transcription–Polymerase Chain Reaction (qRT-PCR) Analysis

The anti-photoaging mechanisms of SFP and DSFP-45 were studied by analyzing the miRNA expression of collagen and pro-inflammatory cytokines in photoaged cells. HaCaT cells were seeded in 6-well plates at 6 × 10^5^ cells per well and treated according to Section 3.8.2. The cells were collected, and the qRT-PCR analysis was performed according to our previous method [67]. Briefly, the total RNA of the cells was extracted with a Trizol reagent (15596026, Invitrogen, Carlsbad, CA, USA). The extracted RNA was then purified and quantified using a micro-spectrophotometer (K5800C, Beijing Kaiao Technology Development Co., Ltd., Beijing, China). The purified RNA was converted into complementary DNA (cDNA) by reverse transcription, which was conducted using the RevertAid First Strand cDNA Synthesis Kit (K1622, Applied Biosystems, Foster City, CA, USA). The qRT-PCR reaction was performed on a CFD-3120 Real-Time PCR detection system (Bio-Rad Laboratories, Inc., Hercules, CA, USA). The gene expression was analyzed using the TSINGKE^®^ Master qPCR Mix (SYBR Green I) (Tsingke Biotechnology Co., Ltd., Beijing, China) by a Mini Opticon^TM^ Detector (CFD-3120, Bio-Rad). The primer sequences of genes are available in Table 4. *GAPDH* was used as an internal control. The relative fold change in gene expression was calculated using the 2^−ΔΔCT^ method.

### 3.9. Statistical Analysis

Statistical analysis was conducted with GraphPad Prism version 8 (GraphPad Software, La Jolla, CA, USA). Means of multiple groups were compared using one-way analysis of variance (ANOVA). Means of two groups were compared using the Student *t*-test. *p* < 0.05 was the accepted significance level. Data were presented as the mean ± standard deviation.

## 4. Conclusions

This study investigated the effects of UV/H_2_O_2_ treatment on the structural characteristics and anti-photoaging activity of *S. fusiforme* polysaccharides. The treatment was an effective way to degrade polysaccharides and enhance their protective effects against UVB irradiation. Among the degraded products, DSFP-45, obtained from UV/H_2_O_2_ treatment for 45 min, exhibited the strongest anti-photoaging activity, improving the cell viability and hydroxyproline level of photoaged HaCaT cells. In addition, DSFP-45 exerted anti-photoaging effects by promoting the production and expression of collagen I and inhibiting the cellular inflammatory response. These findings suggest that UV/H_2_O_2_ treatment for 45 min effectively improved the antagonistic effects of *S. fusiforme* polysaccharides against skin photoaging, and DSFP-45 could be used as a potential therapeutic strategy for skin photoaging. However, the anti-photoaging mechanisms and structure information of DSFP-45 need to be further studied. In the follow-up study, we will investigate the preventive effects and mechanisms of DSFP-45 against skin photoaging in vivo and clarify the precise structure of DSFP-45.

## Figures and Tables

**Table 1:** Chemical compositions of SFP and DSFPs.

	Total Carbohydrate (%)	Protein (%)	Reducing Sugar (%)	Uronic Acids (%)	Sulfate (%)
SFP	50.42 ± 1.09 ^a^	2.72 ± 0.05 ^b^	1.29 ± 0.29 ^a^	35.52 ± 0.47 ^f^	6.14 ± 0.97 ^a^
DSFP-15	52.12 ± 2.64 ^ab^	2.56 ± 0.83 ^ab^	1.41 ± 0.18 ^a^	32.24 ± 0.19 ^d^	5.96 ± 0.47 ^a^
DSFP-30	62.04 ± 2.62 ^c^	1.54 ± 0.22 ^ab^	2.71 ± 0.75 ^b^	34.29 ± 0.35 ^def^	6.26 ± 0.67 ^ab^
DSFP-45	59.44 ± 2.95 ^bc^	1.87 ± 0.47 ^ab^	3.09 ± 0.66 ^bc^	33.70 ± 0.96 ^def^	5.76 ± 0.15 ^a^
DSFP-60	60.40 ± 2.83 ^bc^	1.30 ± 0.50 ^ab^	3.91 ± 0.50 ^c^	34.80 ± 1.80 ^ef^	6.41 ± 0.26 ^ab^
DSFP-75	60.82 ± 2.78 ^bc^	1.36 ± 0.25 ^ab^	5.16 ± 1.07 ^d^	32.92 ± 1.43 ^de^	7.12 ± 0.27 ^bc^
DSFP-90	72.14 ± 3.96 ^d^	1.80 ± 0.44 ^ab^	5.66 ± 0.48 ^d^	29.88 ± 1.16 ^c^	8.15 ± 0.30 ^de^
DSFP-105	71.47 ± 5.99 ^d^	1.31 ± 0.13 ^a^	5.86 ± 0.68 ^d^	22.51 ± 1.16 ^b^	7.46 ± 0.40 ^cd^
DSFP-120	66.51 ± 4.55 ^cd^	1.25 ± 0.27 ^ab^	6.16 ± 0.25 ^d^	19.46 ± 1.42 ^a^	8.81 ± 0.50 ^e^

^a–f^ Means in each column with different superscripts represent significant difference (*p* < 0.05).

**Table 2:** Molecular weights and yields of SFP and DSFPs.

	Molecular Weight (kDa)	Yield (%)
SFP	271	-
DSFP-15	128	65.7
DSFP-30	59	56.0
DSFP-45	43	56.7
DSFP-60	36	48.3
DSFP-75	31	33.3
DSFP-90	28	33.3
DSFP-105	28	38.3
DSFP-120	26	30.0

**Table 3:** Monosaccharide compositions of SFP and DSFPs.

Fraction		Molar Proportion of Monosaccharides (%)				
	Fucose	Galactose	Glucose	Xylose	Galacturonic Acid	Glucuronic Acid
SFP	35.58	15.96	4.19	6.12	17.53	20.62
DSFP-15	36.70	15.84	3.05	5.69	17.20	21.51
DSFP-30	39.73	16.16	2.67	4.91	15.58	20.95
DSFP-45	38.61	17.06	2.98	5.26	14.46	21.62
DSFP-60	41.50	16.86	2.60	5.02	14.29	19.73
DSFP-75	41.70	16.69	2.51	4.89	13.10	21.11
DSFP-90	43.84	16.24	2.20	4.54	11.27	21.91
DSFP-105	45.47	16.14	2.23	4.44	10.57	21.14
DSFP-120	46.04	16.46	2.20	4.11	8.95	22.23

**Table 4:** Primer sequences of genes in qRT-PCR.

Gene	Primer (5′-3′)	
*GAPDH*	Forward	TCCACTGGCGTCTTCACCACCAT
	Reverse	GGAGGCATTGCTGATGATCTTGAGG
*collagen I*	Forward	CAAGGTGTTGTGCGATGACG
	Reverse	TGGTTTCTTGGTCGGTGGG
*IL-1β*	Forward	CTGTACCTGTCCTGCGTGTT
	Reverse	AGACGGGCATGTTTTCTGCT
*IL-6*	Forward	CTGACCCAACCACAAATGC
	Reverse	TCTGAGGTGCCCATGCTAC
*TNF-α*	Forward	GCTGCACTTTGGAGTGATCG
	Reverse	CTTGTCACTCGGGGTTCGAG
